# Childhood chronic condition and subsequent self-reported internalizing and externalizing problems in adolescence: a birth cohort study

**DOI:** 10.1007/s00431-022-04505-9

**Published:** 2022-07-07

**Authors:** Heidi Määttä, Meri Honkanen, Tuula Hurtig, Anja Taanila, Hanna Ebeling, Heli Koivumaa-Honkanen

**Affiliations:** 1 Department of Psychiatry, Lapland Hospital District, P.O. Box 8041, FI-96101 Rovaniemi, Finland; 2 University of Oulu Graduate School UniOGS, University of Oulu, P.O. Box 8000, FI-90014 Oulu, Finland; 3 Haapaniemi Primary School, City of Kuopio, Aseveljenkatu 8, FI-70620 Kuopio, Finland; 4 Research Unit of Clinical Neuroscience, University of Oulu, P.O. Box 8000, FI-90014 Oulu, Finland; 5 PEDEGO Research Unit, University of Oulu, P.O. Box 8000, FI-90014 Oulu, Finland; 6 Clinic of Child Psychiatry, Oulu University Hospital, P.O. Box 5000, FI-90014 Oulu, Finland; 7 Center for Life Course Health Research, University of Oulu, P.O. Box 8000, FI-90014 Oulu, Finland; 8 Institute of Clinical Medicine, Psychiatry, University of Eastern Finland, P.O. Box 1627, FI-70211 Kuopio, Finland; 9 Mental Health and Wellbeing Center, Kuopio University Hospital, P.O. Box 100, FI-70029 Kuopio, Finland

**Keywords:** Chronic condition, Internalizing problems, Externalizing problems, Childhood, Adolescence, Longitudinal study

## Abstract

Chronic conditions are common in childhood. We investigated the associations of childhood chronic conditions reported by parents with subsequent self-reported internalizing and externalizing problems in adolescence. A sample of 6290 children (3142 boys and 3148 girls) with data on chronic condition reported by parents both at 7 and at 16 years of age was obtained from the Northern Finland Birth Cohort 1986 (NFBC 1986), which is a longitudinal 1-year birth cohort (*n* = 9432) from an unselected, regionally defined population. Internalizing and externalizing problems were measured at 8 years of age with Rutter Children’s Behavioral Questionnaire by teachers and at 16 years of age with Youth Self-Report by adolescents. When studying the effects of history of chronic conditions on these problems at 16 years of age, childhood internalizing and externalizing problems and social relations were adjusted. A history of chronic condition predicted subsequent somatic complaints among all adolescents. Early-onset chronic conditions were related to subsequent externalizing (OR 1.35; 1.02–1.79) and attention problems (OR 1.33; 1.01–1.75) and later onset of chronic conditions with internalizing (OR 1.49; 1.22–1.82) and thought problems (OR 1.50; 1.18–1.92). The effect was specific for sex and the type of chronic condition.

*Conclusion*: Childhood chronic conditions predicted internalizing and externalizing problems in adolescence. To prevent poor mental health trajectories, children with chronic conditions during their growth to adolescence need early support and long-term monitoring.

**Table:** 

**What is Known:** *• Childhood adversities increase the risk of mental disorders.* *• Internalizing and externalizing problems have been suggested for measuring childhood and adolescent psychopathologies.*	
**What is New:** *• Having a chronic condition (CC) before the age of 7 or later but before the age of 16 had different outcomes in adolescence. The early onset predicted externalizing problems, whereas the late onset predicted internalizing problems and thought problems in adolescence. The risk of somatic complaints was increased regardless of CC onset time. These findings can reflect more restricted ability to mental processing in the younger children.*

**What is Known:**

*• Childhood adversities increase the risk of mental disorders.*

*• Internalizing and externalizing problems have been suggested for measuring childhood and adolescent psychopathologies.*

**What is New:**

*• Having a chronic condition (CC) before the age of 7 or later but before the age of 16 had different outcomes in adolescence. The early onset predicted externalizing problems, whereas the late onset predicted internalizing problems and thought problems in adolescence. The risk of somatic complaints was increased regardless of CC onset time. These findings can reflect more restricted ability to mental processing in the younger children.*

## Introduction

Chronic conditions (CCs) are the leading cause of disability worldwide, having accounted for up to 60% of the global burden of disease [1]. CCs are defined by at least 1-year duration, a need for ongoing medical care and functional limitation [2–4]. The prevalence of childhood CCs (such as asthma, cerebral palsy, or juvenile rheumatoid arthritis) can vary a great deal (3.5–35%) depending on the source of information, the method of information retrieval, and the study population [4]. CCs among children and adolescents have and will increase [5, 6]. In a large US survey, the end-study prevalence of the three subsequent cohorts was 13% in 1994, 25% in 2000, and 26% in 2006 with substantial turnover with CCs [7].

CCs can threaten one’s physical, social, and psychological well-being, increasing, thus, the risk of mental disorders [8–10]. In addition, it has been suggested that severe, humiliating, or frequent symptoms are prone to be more deleterious to mental health [11–14]. According to Caspi and Moffitt, mental disorders show little causal specificity, morphing within time into other conditions and co-morbidities. Thus, generalized liability to develop psychopathology, comorbidity, and persistence of disorders over time needs dimensional models. They suggested internalizing and externalizing problems for measuring childhood and adolescent psychopathologies [15].

The cross-sectional associations of CCs with externalizing and internalizing problems are widely reported [16–22], the last being more prevalent [17, 19, 20, 23]. CCs are also specifically linked with social, thought, and attention problems [21, 24, 25]. Apart from a few large longitudinal population-based studies [26–28] and some comprehensive meta-analyses [20, 23, 29], the majority had small samples and was performed in clinical settings with severely ill children. Even in adolescence, more often parents’ and teachers’ reports have been studied than adolescents’ own assessments [20, 23].

In our previous study on the longitudinal associations of childhood CCs with adolescents’ self-reported health and life satisfaction, adolescents with CC tended to adapt and be satisfied with their life, but this varied by sex and the age of onset and type of CC [10]. In the present study, we now explored further whether and how childhood CCs predict adolescents’ self-reported internalizing and externalizing problems. We hypothesized that childhood CCs increase the risk of these problems in adolescence, but this varies by the age of onset and type of CC.

## Materials and methods

### Study design

The Northern Finland Birth Cohort 1986 (NFBC 1986) is a longitudinal 1-year birth cohort from an unselected, regionally defined population. It consisted of 9,432 live-born children born between July 1985 and June 1986 in the northernmost Finnish provinces, Oulu and Lapland [30]. Data collection started before the children’s birth with mothers at their first antenatal clinic visit.

*The first follow-up data* was collected at the autumn of the first school year when the children started school and were 7 years old (*C7*). A postal questionnaire was sent to the parents of 9,326 children. Responses were obtained from the parents of 8,416 children (90%). Later in the spring of this first school year, the child being 8 years old (*C8*), the teachers assessed the behavior of 8525 children (92%) by responding to their own questionnaire.

The *second follow-up* was carried out when the children were 16 years old (*A16*). A postal questionnaire to 9,215 adolescents and another for their parents were sent. Responses were obtained from 7,344 adolescents (80%) and from the parents of 6985 adolescents (76%). Children with intellectual disability (*n* = 115) were excluded.

**Fig. 1:**
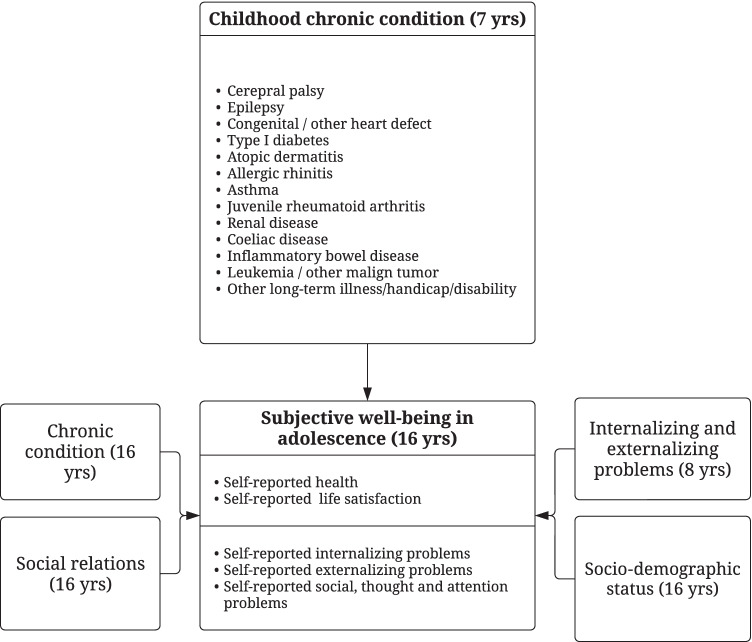
The project on childhood chronic condition and subjective well-being in adolescence

Written informed consent was obtained from the participants. The research plan was approved by the ethical committee of the Northern Ostrobothnia Hospital District. Our study design including our previous works on the subjective well-being in adolescence is presented in Fig. 1.

### Outcome

*Self-reported internalizing and externalizing problems in adolescence* were measured as an outcome at A16 with the widely used and validated 118-item Achenbach’s Youth Self-Report (YSR) [31, 32] to assess competence and problems during the previous 6 months. We used the 1991 version, but data analyses were conducted according to the categorization of the 2001 version [30]. Our questionnaire lacked six items (*cf.* below) from the 2001 version.

YSR has eight problem subscales: (a) *withdrawn/depressed* (7 items; lacking item “enjoys a little”); (b) *somatic complaints* (10 items); (c) *anxious/depressed* (13 items); (d) *social problems* (10 items; lacking item “jealous”); (e) *thought problems* (12 items); f) *attention problems* (7 items; lacking items “fail to finish” and “inattentive”); (g) *delinquent behavior* (12 items; lacking items “breaks rules” and “tobacco”, but including an item on alcohol and drugs); and (h) *aggressive behavior* (17 items). The three response alternatives were “does not apply” (0 points), “applies somewhat or occasionally” (1 point), or “applies very well or often” (2 points). Adolescents who gave responses to all the items of a subscale in our questionnaire were included in its analyses [33].

We calculated sum score for *YSR total problems* including all its eight subscales and sum scores for each of these subscales [33]. *Internalizing problems* include withdrawn, somatic complaints and anxious/depressed subscales. As somatic complaints may considerably bias our analyses of children with CCs [34, 35], we also calculated a sum score for *internalizing problems without somatic complaints*. *Externalizing problems* include delinquent and aggressive behaviors.

The Cronbach’s alpha coefficient for YSR total problems was 0.93, and for internalizing and externalizing problems, it was 0.87, each. In YSR subscales, it ranged 0.62–0.83, being the highest for aggressive behavior (0.83), followed by anxiety/depression (0.81), somatic complaints (0.74), thought problems (0.71), delinquent behavior (0.70), withdrawal/depression (0.66), attention problems (0.63), and social problems (0.62).

The *cut-off for possible psychiatric problems* in the YSR has generally been the 82nd percentile [33]. Thus, the following scores indicated high level of symptoms: withdrawn/depressed ≥ 5 (82.5%); somatic complaints ≥ 7 (85.7%); anxious/depressed ≥ 7 (84.6%); social problems ≥ 4 (81.8%); thought problems ≥ 5 (84.1%); attention problems ≥ 6 (79.0%); delinquent behavior ≥ 6 (79.4%); aggressive behavior ≥ 11 (83.9%); internalizing problems with somatic complaints ≥ 15 (80.3%) or without ≥ 10 (80.5%); externalizing problems ≥ 15 (79.9%); and YSR total problems (81.9%).

### Chronic conditions as explanatory variables

The *longitudinal data on CCs* were obtained from parents of 8036 children at C7 and parents of 6680 adolescents at A16 by questions (one with open-end) focusing on long-term illnesses, disabilities, and handicaps. More detailed information (i.e., prevalence by sex and type of CC) has been given in our previous report [10]. In the analyses, CCs were divided into four age-related groups:

1) Early onset: CC (+ / +) present both C7 and A16 (*n* = 494)

2) Recovered: CC ( + / -) present at C7 but not at A16 (*n* = 411)

3) Late onset: CC (- / +) not present at C7 but present at A16 (*n* = 767)

4) No CC: CC (- /-) present neither at C7 nor A16 (*n* = 4618)

The *data on both data collection times* were obtained from 6,290 children, and of them, YSR subscales at A16 were available as follows: withdrawn/depressed (*n* = 5,579), somatic complaints (*n* = 5,596), anxious/depressed (*n* = 5,367), social problems (*n* = 5,679), thought problems (*n* = 5,400), attention problems (*n* = 5,725), delinquent behavior (*n* = 5,620), and aggressive behavior (*n* = 5,534). Internalizing problems with (*n* = 5,074) and without (*n* = 5,256) somatic complaints, externalizing problems (*n* = 5,381), and YSR total problems (*n* = 4,478) were calculated.

*Type of CC at C7* was obtained from 6,513 children with atopic/asthmatic conditions (i.e., atopic dermatitis, allergic rhinitis, asthma) being most common (12.7%) type of CC [10]. Children having both atopic/asthmatic CC and any other CC (*n* = 16) at C7 were excluded from these numbers due to the small sample size. In the analyses, types of CC at C7 were divided into three groups: atopic/asthmatic CC (*n* = 818); other CC (*n* = 310); and no CC (*n* = 5,385).

In these, data on YSR subscales was available as follows: withdrawn/depressed (*n* = 5,640), somatic complaints (*n* = 5,660), anxious/depressed (*n* = 5,424), social problems (*n* = 5,741), thought problems (*n* = 5,454), attention problems (*n* = 5,788), delinquent behavior (*n* = 5,680), and aggressive behavior (*n* = 5,594). Internalizing problems with (*n* = 5,127) and without (*n* = 5,310) somatic complaints, externalizing problems (*n* = 5,437), and YSR total problems (*n* = 4,518) were calculated.

### Other possible confounding factors

*Internalizing and externalizing problems at C8* were assessed by teachers with the widely used and valid 26-item Rutter Children’s Behavioral Questionnaire for teachers (RB2) [36, 37]. As these RB2 problems at C8 can bias the effect of age- and type-related CCs on the YSR internalizing and externalizing problems at A16, we adjusted RB2 problems in all these analyses.

*Socio-demographic status of the family at A16* was obtained from the parents’ data. They included family type (intact, divorced, reconstructed, single parent), social status (professional, skilled worker, unskilled worker, farmer) defined by the parent with the highest social status, annual family income (lowest, middle, or highest third), and number of children in the family, i.e., sibship size (1 child, 2–4 children, 5–10 children, or 10–20 children).

*Self-reported social relations at A16* were measured with four variables, after dichotomization due to highly skewed distribution [38].

1) *Time spent with family*: “How often do you socialize with your family?” The response alternatives were dichotomized (never, seldom or monthly vs. weekly or daily).

2) *Perceived parental interest*: “Are your parents interested in your school, hobbies and other things that matter to you?” The response alternatives were dichotomized in the analyses (never or seldom vs. almost always).

3) *Time spent with friends:* “How often do you meet your friends?” The response alternatives were dichotomized (never, seldom or monthly vs. weekly or daily).

4) *Number of close friends*: “Do you have a close friend with whom you can confidentially discuss your matters?” The response alternatives were dichotomized (no close friends vs. at least one close friend).

### Statistical analyses

The IBM^®^ SPSS^®^ Statistics (version 28) for Mac was used for statistical analyses. In our previous study with the same cohort, we found no significant differences between age- or type-related CC groups with respect to sex, family type, social status, annual income, sibship size, time spent with family, or perceived family time [10]. Thus, we now used the Pearson Chi-square test to investigate further these associations with the number of close friends and time spent with friends as well as with RB2 at C8. Similarly, we used it also to study characteristics of the adolescents by YSR internalizing and externalizing problems at A16 (Table 1).

**Table 1:** Characteristics of the adolescents by Youth Self-Report (YSR) internalizing and externalizing problems

Characteristics at A16	YSR internalizing problems at A16									YSR externalizing problems at A16							
	No			Yes			Test statistic			No			Yes			Test statistic	
	*n*	*%*		*n*	*%*	*χ*^*2*^		p^*^		*n*	*%*		*n*	*%*	*χ*^*2*^		p^*^
Sex																	
Male	2615	92.0		228	8.0	464.44		< 0.001		2532	83.7		494	16.3	50.716		< 0.001
Female	2192	69.8		948	30.2					2547	76.5		782	23.5			
Family type																	
Intact	3095	81.9		682	18.1	17.048		0.001		3245	81.8		724	18.2	29.617		< 0.001
Divorced	496	77.0		148	23.0					534	76.5		164	23.5			
Reconstructed	350	75.8		112	24.2					365	73.3		133	26.7			
Single parent	29	76.3		9	23.7					34	70.8		14	29.2			
Family social status																	
Professional	3389	81.0		796	19.0	2.898		0.408		3576	80.5		865	19.5	7.605		0.055
Skilled worker	686	79.2		180	20.8					711	77.2		210	22.8			
Unskilled worker	38	82.6		8	17.4					41	83.7		8	16.3			
Farmer	165	77.5		48	22.5					190	83.7		37	16.3			
Annual family income																	
Lowest third	1076	77.5		313	22.5	12.076		0.002		1157	77.2		342	22.8	10.949		0.004
Middle third	1208	82.6		254	17.4					1251	81.4		286	18.6			
Highest third	1195	80.5		289	19.5					1270	81.3		292	18.7			
Sibship size																	
1 child	816	81.3		188	18.7	0.571		0.903		859	79.5		221	20.5	28.630		< 0.001
2–4 children	2846	80.3		698	19.7					2950	78.8		793	21.2			
5–10 children	471	81.1		110	18.9					531	85.8		88	14.2			
11–20 children	88	80.7		21	19.3					107	93.0		8	7.0			
Time spent with family																	
Monthly at most	975	75.4		318	24.6	25.427		< 0.001		952	68.5		437	31.5	143.31		< 0.001
Weekly at least	3801	81.7		851	18.3					4090	83.1		831	16.9			
Perceived parental interest																	
Seldom or never	606	70.8		250	29.2	57.695		< 0.001		632	68.0		298	32.0	96.165		< 0.001
Often	4167	81.9		918	18.1					4405	81.9		972	18.1			
Time spent with friends																	
Monthly at most	215	67.4		104	32.6	35.960		< 0.001		306	85.7		51	14.3	7.981		0.005
Weekly at least	4580	81.1		1067	18.9					4756	79.5		1223	20.5			
Number of close friends																	
No close friends	315	77.8		90	22.2	1.803		0.179		352	80.5		85	19.5	0.119		0.730
At least one close friend	4449	80.5		1076	19.5					4676	79.9		1179	20.1			

^*^Pearson Chi-square test; in all: degree of freedom = 1

Internal consistency of YSR was measured with Cronbach’s alpha. Multinomial logistic regression with odds ratios (OR) and 95% confidence intervals (CI) was used to explore the independent association of age- and type-related CCs (reference group for both: no CC) with YSR at A16. The fully adjusted models included the following covariates: sex, family type, sibship size, annual family income, time spent with family, perceived parental interest, and time spent with friends. The Hosmer and Lemenshow test was used to measure the overall fit of the logistic model.

## Results

### Characteristics of adolescents by CCs, RB2, YSR, and loss analyses

There were no significant differences between age- or type-related CC groups with respect to time spent with friends and number of close friends at A16 or RB2 internalizing and externalizing problems assessed by teachers at C8.

YSR internalizing and externalizing problems were associated with female sex, non-intact family type, the lowest third annual family income, and low self-reported social relations with the family (i.e., less time spent with family and less perceived parental interest) at A16. Less time spent with friends was associated with internalizing problems, whereas more such time with externalizing problems. Large sibship size seemed to be protective against externalizing problems (Table 1).

According to loss analyses, there were no differences in CC status between responders and non-responders with respect to YSR. The non-responders were more often boys, lived less often with both parents, and had parents with lower education and lower employment status than responders.

### Age- and type-related CCs as predictors for YSR

YSR internalizing problems, with and without somatic complaints, as well as specific YSR subscales (i.e., anxiety/depression, thought problems, and somatic complaints) were associated with CC status. These problems were most common among adolescents with CC (- / +) and CC (+ / +). Furthermore, YSR total problems differed significantly according to CC status (Table 2).

**Table 2:** Youth Self-Report (YSR) mental problems by age-related chronic condition (CC)

Youth Self-Report	Age-related CC													
	CC (+ / +)^a^			CC ( + / -)^b^			CC (- / +)^c^			CC (- / -)^d^			Test statistic	
	*n*	*%*		*n*	*%*		*n*	*%*		*n*	*%*	*χ*^*2*^		p^*^
Withdrawn/depressed	80	18.7		55	15.0		139	20.1		677	16.5	7.231		0.065
Somatic complaints	70	16.6		56	15.2		115	16.7		530	12.9	11.296		0.010
Anxious/depressed	71	17.4		44	12.8		122	18.4		578	14.6	9.317		0.025
Social problems	94	21.4		63	17.0		135	19.3		746	17.9	4.243		0.236
Thought problems	65	15.5		45	12.8		133	20.0		594	15.0	12.934		0.005
Attention problems	99	22.0		64	17.2		149	21.3		860	20.5	3.411		0.332
Delinquent behavior	95	22.3		68	18.7		152	22.0		803	19.4	4.502		0.212
Aggressive behavior	83	19.3		50	14.1		98	14.4		643	15.8	5.607		0.132
Internalizing problems	87	23.1		60	18.0		157	24.8		676	18.1	19.627		< 0.001
Internalizing problems without somatic complaints	88	22.2		60	17.5		158	24.2		712	18.4	14.757		0.002
Externalizing problems	93	22.7		66	19.1		136	20.5		767	19.3	2.975		0.396
YSR total problems^e^	76	22.7		53	18.5		122	21.6		578	17.6	15.745		0.023

^a^Chronic condition both at 7 and at 16 years of age

^b^Chronic condition at 7 but not at 16 years of age

^c^Chronic condition at 16 but not at 7 years of age

^d^No chronic condition at 7 or at 16 years of age

^e^YSR total problems included all eight YSR subscales

^*^Pearson Chi-square test; in all: degree of freedom = 1

In *fully adjusted logistic regression analyses* with CC (- / -) as reference group, CC (+ / +) was associated with internalizing (OR 1.64; 1.20–2.23) and externalizing problems (OR 1.35; 1.02–1.79) and YSR total problems (OR 1.76; 1.23–2.52) at A16. When YSR subscales were separately analyzed, also the risk of somatic complaints, attention problems, and delinquent and aggressive behavior were increased (Table 3). Similarly analyzed, CC (- / +) was associated with higher risk for internalizing problems with (OR 1.69; 1.33–2.14) and without somatic complaints (OR 1.54; 1.22–1.94) and total problems (OR 1.65; 1.25–2.19), but not with externalizing problems. In subscale analyses, CC (- / +) group had a higher risk of thought problems, somatic complaints, anxiety/depression, and withdrawal/depression subscales, whereas adolescents with CC ( + / -) had higher risk only of somatic complaints (OR 1.49; 1.04–2.14) (Table 3).

**Table 3:** The risk of having self-reported mental problems measured with Youth Self-Report (YSR) in adolescence (A16) according to age-related chronic condition (CC), the reference group being subjects with no CC at any age

Youth Self-Report	Age-related CC*							
	CC (+ / +)^a^			CC ( + / -)^b^			CC (- / +)^c^	
	Crude OR	Adjusted** OR		Crude OR	Adjusted** OR		Crude OR	Adjusted** OR
**Withdrawn/depressed**	1.16 (0.90–1.50)	1.12 (0.82–1.53)		0.89 (0.66–1.20)	0.93 (0.66–1.34)		**1.27 (1.04**–**1.56)**	**1.30 (1.02**–**1.64)**
**Somatic complaints**	**1.35 (1.03**–**1.77)**	**1.67 (1.20**–**2.32)**		1.22 (0.90–1.64)	**1.49 (1.04**–**2.14)**		**1.36 (1.09**–**1.69)**	**1.43 (1.10**–**1.87)**
**Anxious/depressed**	1.23 (0.94–1.61)	1.33 (0.95–1.85)		0.85 (0.61–1.18)	0.89 (0.60–1.32)		**1.31 (1.06**–**1.63)**	**1.38 (1.07**–**1.79)**
**Social problems**	1.25 (0.98–1.59)	1.25 (0.93–1.67)		0.94 (0.71–1.25)	0.93 (0.66–1.31)		1.10 (0.90–1.35)	1.22 (0.97–1.55)
**Thought problems**	1.04 (0.79–1.38)	1.12 (0.81–1.56)		0.83 (0.60–1.15)	1.01 (0.70–1.46)		**1.42 (1.15**–**1.74)**	**1.50 (1.18**–**1.92)**
**Attention problems**	1.10 (0.87–1.39)	**1.33 (1.01**–**1.75)**		0.81 (0.61–1.07)	0.91 (0.65–1.27)		1.05 (0.86–1.28)	1.15 (0.91–1.44)
**Delinquent behavior**	1.19 (0.94–1.52)	**1.44 (1.09**–**1.90)**		0.96 (0.73–1.26)	1.07 (0.77–1.47)		1.17 (0.97–1.43)	1.20 (0.95–1.50)
**Aggressive behavior**	1.27 (0.99–1.64)	**1.38 (1.03**–**1.86)**		0.88 (0.64–1.20)	0.92 (0.64–1.33)		0.90 (0.71–1.13)	0.86 (0.66–1.13)
**Internalizing problems**^**d**^	**1.36 (1.05**–**1.75)**	**1.64 (1.20**–**2.23)**		0.99 (0.74–1.33)	1.18 (0.83–1.67)		**1.49 (1.22**–**1.82)**	**1.69 (1.33**–**2.14)**
**Int. problems without**^**e**^	1.27 (0.99–1.63)	1.34 (0.98–1.81)		0.94 (0.71–1.26)	1.02 (0.72–1.44)		**1.41 (1.16**–**1.72)**	**1.54 (1.22**–**1.94)**
**Externalizing problems**	1.22 (0.96–1.56)	**1.35 (1.02**–**1.79)**		0.99 (0.75–1.30)	1.11 (0.80–1.54)		1.06 (0.83–1.34)	1.06 (0.83–1.34)
**YSR total problems**^**f**^	**1.38 (1.05**–**1.81)**	**1.76 (1.23**–**2.52)**		1.07 (0.78–1.45)	1.10 (0.71–1.70)		**1.30 (1.04**–**1.61)**	**1.65 (1.25**–**2.19)**

^a^Chronic condition both at 7 and at 16 years of age

^b^Chronic condition at 7 but not at 16 years of age

^c^Chronic condition at 17 but not at 7 years of age

^d^Internalizing problems with somatic complaints

^e^Internalizing problems without somatic complaints

^f^YSR total problems included all eight YSR subscales

*Reference category: No chronic condition at 7 or at 16 years of age

**Adjusted for sex, family type, annual family income, sibship size, time spent with family, perceived parental interest, time spent with friends

Bold entries reached the level of statistical significance (*p* < 0.05)

When the analyses were made *separately by sex*, CC (+ / +) was associated with increased odds of somatic complaints (OR 2.02; 1.34–2.93) and delinquent behavior (OR 1.52; 1.03–2.27) only among girls, and CC ( + / -) with somatic complaints (OR 2.20; 1.17–4.12) only among boys. CC (- / +) was associated with somatic complaints (OR 1.38; 1.02–1.86) and anxiety/depression (OR 1.42; 1.06–1.89) only among girls, but with thought problems regardless of sex (OR_girl_ 1.37; 1.02–1.85 and OR_boy_ 1.87; 1.23–2.84).

When the most common type of CCs, i.e., *atopic/asthmatic CCs*, were separately analyzed, adolescents with atopic/asthmatic CC at C7 were at higher risk for internalizing problems (OR 1.34; 1.03–1.75), somatic complaints (OR 1.58; 1.19–2.09), and delinquent behavior (OR 1.28; 1.00–1.63) compared to adolescents without a history of CC. Other CCs at C7, when separately analyzed, were not associated with subsequent YSR.

In all the analyses, *further adjustments with RB2 at C8*–i.e., internalizing and externalizing problems assessed by the child’s teacher–did not alter the risks.

## Discussion

The large prospective population-based 1-year birth cohort (NFBC 1986) showed that CCs during the growth from childhood to adolescence were associated with the adolescent’s somatic complaints. Children with the early onset of CCs had also over the 1.3-fold higher risk of self-reported externalizing and attention problems in adolescence, whereas children with late-onset CCs had the 1.5-fold higher risk both on self-reported internalizing and thought problems than children with no CCs. The increased risk of these problems was shown even after controlling for their presence already in childhood.

Despite the large number of studies on the cross-sectional associations between childhood CC and mental health, the effect of CC onset age on its consequences has remained sparsely studied. In a previous study [39], it was suggested that childhood cancer survivors more often need later hospital contacts for mental disorders than their healthy controls, the risk being related to age of onset, not to time since diagnosis. The risk was particularly high among children under the age of 10 at the time of diagnosis. A recent study [40] also showed that children with CCs presented an excessive rate of psychiatric illness already at the age of 10, and these CCs continued to be associated with poor mental health outcomes at the age of 13 and 15.

In the present study, the follow-up time, the exposure (CCs), and/or the outcome (YSR) had a broader range than these previous studies. The outcome included not only severe conditions needing mental hospitalization but also self-reported internalizing and externalizing problems. Based on our results on large birth cohort of over 9000 children followed from the age of 7 to the age of 16, chronic condition was a challenge for a child regardless of the age of onset. Further, only childhood CC with recovery before the age of 16 appeared to predict solely somatic complaints, but not mental problems broadly in adolescence.

In addition to the age of onset, both sex and the type of CC play a role. In the present study, only girls with early-onset CC were at a risk of delinquent behavior. This result differs from those of previous studies [20], in which externalizing problems were more characteristic of boys with CC. Instead, in line with the previous studies [41, 42] was our finding that children with atopic/asthmatic CC were at an increased risk of somatic complaints and delinquent behavior in adolescence. This is important finding due to high prevalence of atopic and asthmatic conditions [10, 43].

However, personal strengths and abilities to cope and adjust can buffer the effects of CCs [3, 18, 44]. A child’s ability to adapt to adversities depends on personal factors, such as temperament, level of maturity, and cognitive skills [45, 46]. Age-specific psychological adaptation mechanisms play a role in development of externalizing [47, 48] and internalizing problems [49] and seem to explain also the differences in YSR profiles between the early and late onset age of CC. Furthermore, the stage of brain development during stress due to the onset of CC may have an impact on future psychopathology [50].

In addition, negative feelings (e.g., uncertainty, frustration, guilt, and exhaustion), which are common among parents with chronically ill children [51–55], impair parents’ capability to provide support for their chronically ill children. Furthermore, children and adolescents with CC might be at an increased risk of peer victimization [40, 56] and social problems [20], but this was not shown in our study. Instead, time spent with family and friends was protective against internalizing and externalizing problems of adolescents in our study.

Finally, it is important to notice that all adolescents with a history of CC reported somatic complaints more often than adolescents without it, even if the CC was no longer prevalent in adolescence. This parallels with our previous study on CCs increasing the risk of poor self-reported health [10]. Children and adolescents with a history of CCs may have difficulties to differentiate organ- and stress-related symptoms. This should be acknowledged when evaluating somatic complaints among children and adolescents with CCs [34, 35].

The strengths of the present study include a large, unselected population with a high participation rate and with prospective data on CCs. Self-reported internalizing and externalizing problems were examined both with combined scales and in detail with eight YSR subscales providing a broad picture of the studied associations. Furthermore, we adjusted several important covariates such as socio-demographic status, social relations, and internalizing and externalizing problems assessed by teachers already at the child’s age of eight.

There were also limitations in our study. The information about CCs was based on parents’ reports, not on verified diagnoses, but at least severe conditions were most probably well-known and correctly reported by the parents. Further, data on the type of CC in adolescence was less disease specific than in childhood, and it is possible that children with early-onset CC might have suffered from different conditions in childhood and adolescence. However, only some chronic illnesses have shown type-specific relationships with psychiatric illnesses [57]. Despite these limitations, the present study provides important prospective data on the consequences of CCs since childhood with respect to mental health in adolescence.

## Conclusions

Regardless the age of onset of childhood CCs or its recovery, it increases the risk of somatic complaints in adolescence. In general, the early onset predicted externalizing problems, and the late onset predicted internalizing problems and thought problems. These findings can reflect the more restricted ability to mental processing in the younger children. To prevent poor mental health trajectories, children and adolescents with CC need early support and long-term monitoring during their growth.

## Data Availability

The data from the Northern Finland Birth Cohort research program for health and well-being can be requested for research collaboration projects with the University of Oulu.

## Abbreviations

*A16*: 16 years old
*C7*: 7 years old
*C8*: 8 years old
*CC*: Chronic condition
*CCs*: Chronic conditions
*CI*: Confidence interval
*OR*: Odds ratio
*RB2*: Rutter B2
*YSR*: Youth Self-Report
